# Environmental detection and spreading of mpox in healthcare settings: a narrative review

**DOI:** 10.3389/fmicb.2023.1272498

**Published:** 2023-12-21

**Authors:** Amira Mohamed Taha, Basant E. Katamesh, Abdul Rhman Hassan, Omar Ahmed Abdelwahab, Sarvesh Rustagi, Dang Nguyen, Kenneth Silva-Cajaleon, Alfonso J. Rodriguez-Morales, Aroop Mohanty, D. Katterine Bonilla-Aldana, Ranjit Sah

**Affiliations:** ^1^Faculty of Medicine, Fayoum University, Fayoum, Egypt; ^2^Medical Research Group of Egypt (MRGE), Negida Academy, Arlington, MA, United States; ^3^Faculty of Medicine, Tanta University, Tanta, Egypt; ^4^Mayo Clinic, Rochester, MN, United States; ^5^Faculty of Medicine, Al-Azhar University, Cairo, Egypt; ^6^School of Applied and Life Sciences, Uttaranchal University, Dehradun, India; ^7^Massachusetts General Hospital, Corrigan Minehan Heart Center and Harvard Medical School, Boston, MA, United States; ^8^Faculty of Environmental Sciences, Universidad Científica del Sur, Lima, Peru; ^9^Grupo de Investigación Biomedicina, Faculty of Medicine, Fundación Universitaria Autónoma de lasAméricas-Institución Universitaria Visión de las Américas, Pereira, Colombia; ^10^Gilbert and Rose-Marie Chagoury School of Medicine, Lebanese American University, Beirut, Lebanon; ^11^Department of Clinical Microbiology, All India Institute of Medical Sciences, Gorakhpur, India; ^12^Research Unit, Universidad Continental, Huancayo, Peru; ^13^Tribhuvan University Teaching Hospital, Kathmandu, Nepal; ^14^Department of Clinical Microbiology, DY Patil Medical College, Hospital and Research Centre, DY Patil Vidyapeeth, Pune, India

**Keywords:** Mpox, contamination, surface, hospital, epidemiology

## Abstract

Monkeypox virus (MPXV), which causes Monkeypox (Mpox), has recently been found outside its usual geographic distribution and has spread to 117 different nations. The World Health Organization (WHO) designated the epidemic a Public Health Emergency of International Concern (PHEIC). Humans are at risk from MPXV’s spread, which has raised concerns, particularly in the wake of the SARS-CoV-2 epidemic. The risk of virus transmission may rise due to the persistence of MPXV on surfaces or in wastewater. The risk of infection may also increase due to insufficient wastewater treatment allowing the virus to survive in the environment. To manage the infection cycle, it is essential to investigate the viral shedding from various lesions, the persistence of MPXV on multiple surfaces, and the length of surface contamination. Environmental contamination may contribute to virus persistence and future infection transmission. The best possible infection control and disinfection techniques depend on this knowledge. It is thought to spread mainly through intimate contact. However, the idea of virus transmission by environmental contamination creates great concern and discussion. There are more cases of environmental surfaces and wastewater contamination. We will talk about wastewater contamination, methods of disinfection, and the present wastewater treatment in this review as well as the persistence of MPXV on various environmental surfaces.

## 1 Introduction

### 1.1 Aetiology

The zoonotic viral disease monkeypox (Mpox), endemic in central and western Africa, has recently spread to numerous nations in both endemic and nonendemic regions. Over 92,048 cases had been reported up to December 2023 across 117 countries, 110 (~94%) of which have not historically reported mpox. Its incubation period varies from a few days to 3 weeks. Unlike the smallpox virus, the monkeypox virus (MPXV) has a wide range of animal reservoirs that spread it to humans sporadically (Ježek et al., 1987; Di Giulio and Eckburg, 2004; CDC, 2022; Kaler et al., 2022; Moore and Zahra, 2022). Its prodromal symptoms, which initially resemble the flu, are followed by lymphadenopathy and a rash on the face before spreading throughout the body.

However, the clinical picture in the current outbreak is abnormal; according to research done on 23 patients, 95% had a rash, almost two-thirds had 10 or more lesions, 73% had anogenital lesions, and 41% had mucosal lesions (Ladnyj et al., 1972; Learned et al., 2005; Beer and Rao, 2019; Thornhill et al., 2022a).

Additionally, roughly 10% of patients had a single genital ulcer, and others had many lesions at once (Thornhill et al., 2022a). The majority of the time, it is a self-limiting disease that goes away 3–4 weeks after the first signs and symptoms arise, with crust formation and desquamation occurring over the next 7–14 days. After the lesion has healed, there may be light scarring that later darkens.

### 1.2 Epidemiology

Although Mpox outbreaks have grown since their initial detection, they were primarily confined to Africa. In 2003, the United States of America (USA) reported the first cases outside of Africa, with 53 with mpox confirmed infections. United Kingdom (UK) health officials confirmed the Mpox case of a tourist who returned from Nigeria in May 2022. Since then, recorded cases have grown in other nations, including those without a history of travel to endemic regions, but through community transmission, with only a few cases involving women and children. In contrast, most cases involved having sex with men (MSM) (CDC, 2003a; Isidro et al., 2022; Kipkorir et al., 2022; Kozlov, 2022; Mahase, 2022; Americo et al., 2023).

### 1.3 MPXV clades

Central African and western African are the two primary clades of MPXV, and they are now referred to as clades I and II, respectively. With a case fatality rate of up to 10%, clade I is more severe than clade II, which is less severe with a case fatality rate of less than 1%. Clade II would be divided further into clades IIa and IIb. The MPXV responsible for the current outbreak is related to clade IIb, which is related to clade IIa, responsible for the outbreak in Nigeria in 2017.

That is determined through phylogenetic analysis. It is uncertain what genetic variations are responsible for the greater incidence of clade IIb transmission in humans (Gammon et al., 2010; Isidro et al., 2022; Kipkorir et al., 2022; Kozlov, 2022; Mahase, 2022; Americo et al., 2023). DNA viruses like MPXV typically do not show many mutations. However, in the current outbreak, isolates from 2022 were discovered to have 10 alterations in the MPXV replication complex (RC) and other viral proteins. The potential effects of each mutation are not now fully understood. More study is required to understand further how these recently generated mutations function (Arita et al., 1985; Ježek et al., 1988a; CDC, 2003a; Hutson et al., 2009; Gammon et al., 2010; Isidro et al., 2022; Kannan et al., 2022; Kipkorir et al., 2022; Kozlov, 2022; Mahase, 2022; Americo et al., 2023).

### 1.4 MPXV transmission

Animal interaction was the leading cause of human infection. However, it could be challenging to pinpoint the precise animal interaction that caused a case in areas where different species are encountered. Rarely in secondary cases, but occasionally in primary human patients, does Human-to-human transfer happen. The evidence suggests that family members or those who care for a Mpox patients are at a higher risk for contracting an infection, even though the efficiency of Mpox human-to-human transmission appears to be less than that of smallpox. It did occur up to 11.7% of household contacts of patients who were not protected against smallpox (Arita et al., 1985; Ježek et al., 1988a; Goldmann, 2000; Hutson et al., 2009; Gammon et al., 2010; Spicknall et al., 2010; Kannan et al., 2022; León-Figueroa et al., 2022; Salvato et al., 2022; Hernaez et al., 2023; Karagoz et al., 2023; CDC, 2023a).

The environment, the virus’s ability to infect the host, viral shedding, the host’s susceptibility, and behavior all impact the virus’s spread. Additional essential environmental elements that affect transmission include host density and mobility patterns. Lesion exudate, crust material, respiratory droplets, and viral shedding in faeces are all possible routes for human transmission (Goldmann, 2000; León-Figueroa et al., 2022; Salvato et al., 2022; Atkinson et al., 2022a; Hernaez et al., 2023; Karagoz et al., 2023; Mellon et al., 2023; Sharkey et al., 2023; CDC, 2023a).

It is most likely spread through intimate contact with an infected person and possibly transmitted by big droplets that settle after about one meter; however, the evidence of respiratory transmission is still questionable (Goldmann, 2000; Salvato et al., 2022; Atkinson et al., 2022a; Hernaez et al., 2023). Mpox is spread by close, personal contact, mainly through sexual contact in the current outbreak. Indirect contact with contaminated surfaces and objects is another route that could contribute to viral transmission. A recent investigation in the UK revealed the presence of MPXV DNA on several surfaces from locations visited by a tourist returning from Nigeria with a confirmed case of Mpox (Atkinson et al., 2022a). According to a different investigation, fomite transmission was regarded as a plausible primary infection source in two cases. These studies support environmental assessments since polluted fomite can spread infectious diseases. Fomites might contract the virus by coming into intimate contact with human fluids or secretions, touching contaminated hands, or contacting respiratory droplets that land on various surfaces (Lu et al., 2022; Palich et al., 2022; Atkinson et al., 2022a; Girón-Guzmán et al., 2023; Hernaez et al., 2023; La Rosa et al., 2023; Mellon et al., 2023; Sharkey et al., 2023; CDC, 2023b). Despite patients wearing surgical masks, a different investigation found viral DNA in the air in the outpatient consultation room during visits from Mpox patients. Therefore, the viral particles found may have been transmitted through the air from the skin, sores on the genitalia or oropharynx, or respiratory secretions. Despite this, neither healthcare professionals nor consulting Mpox-negative patients reported any MPXV symptoms during the trial period or in the 21 days that followed (Mellon et al., 2023). Infections of the Mpox virus are transmitted and closely watched due to wastewater (Girón-Guzmán et al., 2023; La Rosa et al., 2023; Sharkey et al., 2023). Since human fluids that are contaminated with the virus may be present in wastewater, it is possible that MPXV can spread there. Therefore, public health officials’ wastewater analysis can provide a clear picture of the scope of the Mpox outbreak and potentially aid in its containment (Ježek et al., 1988b; Nakoune et al., 2017; Adler et al., 2022; Ferré et al., 2022; Lu et al., 2022; Ogoina and Yinka-Ogunleye, 2022; Palich et al., 2022; Riopelle et al., 2022; Thornhill et al., 2022b; Owhonda et al., 2023; Wieder-Feinsod et al., 2023; CDC, 2023b,c).

The possibility of cross-border diffusion and subsequent transmission of Mpox has increased due to the ambiguity surrounding the outbreak’s containment and the risk of social transmission.

Unlike earlier cases, most current cases do not have confirmed travel connections to endemic regions, indicating a crucial role in community and environmental transmission. That review covers the most recent research on the role of the environment in the transmission of Mpox, the importance of wastewater surveillance and monitoring, and the potential transmission of illnesses pathways (Ježek et al., 1988b; Adler et al., 2022; Lu et al., 2022; Palich et al., 2022; Thornhill et al., 2022b; Girón-Guzmán et al., 2023; La Rosa et al., 2023; Sharkey et al., 2023; Wieder-Feinsod et al., 2023; CDC, 2023b,c).

## 2 Viral shedding

Identifying viral shedding to improve treatment and stop further transmission is essential. Because infected skin lesions have the largest viral loads, the highest favorable rates, and the least invasive, quantitative real-time polymerase chain reaction (qPCR) techniques, have been suggested for detecting MPXV in these infected skin lesion swabs. These assessments are most effective when combined with clinical and epidemiological data, such as vaccination history (Ježek et al., 1988b; Adler et al., 2022; Lu et al., 2022; Ogoina and Yinka-Ogunleye, 2022; Palich et al., 2022; Riopelle et al., 2022; Thornhill et al., 2022b; Girón-Guzmán et al., 2023; La Rosa et al., 2023; Owhonda et al., 2023; Wieder-Feinsod et al., 2023; CDC, 2023b,c).

MPXV was found in various swabs, including anal, contaminated objects, skin, saliva, oropharynx, conjunctiva, vaginal fluids, breast milk, semen, blood, urine, and faeces. Given that the viral load in diverse clinical samples coincided with the quantity of replication-competent MPXV found, a higher viral concentration, as assessed by qPCR, can forecast a larger potential for infectivity. After the sickness first appears, the virus might shed for up to 3 weeks. However, it persisted longer in samples with cycle threshold (Ct) values higher than 35, lasting up to 8 weeks for semen and up to 10 weeks for saliva samples, and 6 to 10 weeks for respiratory samples (Ježek et al., 1986, 1988b; Nakoune et al., 2017; Ogoina et al., 2019; Vaughan et al., 2020; Adler et al., 2022; Ferré et al., 2022; Ogoina and Yinka-Ogunleye, 2022; Riopelle et al., 2022; Zachary and Shenoy, 2022; Thornhill et al., 2022b; Owhonda et al., 2023; Wieder-Feinsod et al., 2023).

Numerous diagnostic procedures, such as qPCR, immunohistochemistry, serology, and electron microscopy, could be used to determine the presence of Mpox. The best method for experimental diagnosis is known to be qPCR. However, in an outbreak like this, it may not always be practical because every diagnostic process has to adhere to strict diagnostic and biosafety performance standards regarding sampling technique, storage, and other biosafety requirements, which delays the diagnostic process and causes additional costs. Furthermore, it only has a limited impact in rural places with few resources. Given the rising prevalence of Mpox, quick screening techniques, like those found in SARS-CoV-2 kits, should be created to be employed as a large-scale diagnostic approach for screening and detecting vulnerable people (Ježek et al., 1986; CDC, 2003b; Nakoune et al., 2017; Ogoina et al., 2019; Vaughan et al., 2020; Gould et al., 2022; Morgan et al., 2022; Nörz et al., 2022; Ogoina and Ogunsola, 2022; Pfeiffer et al., 2022; Zachary and Shenoy, 2022).

CDC stated that a patient is no longer contagious when all scabs have gone off, contradicting the traditional view that Mpox patients are infectious until all lesions have crusted. Recent research has shown that MPXV DNA can still be found in the upper respiratory system days after lesions have healed. It is uncertain whether patients with crusted skin lesions and positive upper respiratory tract swabs are contagious. Further study is needed in this area because the healing of skin lesions served as the leading indicator of the Mpox patients’ infectiousness. These findings might prompt additional adjustments to the rules for discharge and quarantine days, with immediate implications for using healthcare resources (CDC, 2003b; Gould et al., 2022; Morgan et al., 2022; Nörz et al., 2022; Ogoina and Ogunsola, 2022; Pfeiffer et al., 2022; Atkinson et al., 2022a,b).

Misdiagnosis of Mpox as another sexually transmitted disease (STD) can delay isolation and result in inadequate treatment, prolonging and worsening the current epidemic. According to several studies, misdiagnosis occurred due to the clinical presentation, which was unusual compared to African outbreaks. A case report showed an atypical distribution of rash that had not happened on the face, in contrast to the recognized national case definition. Only 11% of patients in the UK experienced the rash, the primary diagnostic sign of Mpox, and 20% did not share a prodrome before the rash. Clinical symptoms that are distinct from those that have previously been documented may be the result of an atypical method of transmission. A recent study found that during the 2017–2018 Nigerian outbreak, risky sexual conduct was highly prevalent and that 81.2% of Mpox-infected patients had genital ulcers. Due to this abnormal presentation of Mpox, healthcare professionals may have incorrectly diagnosed Mpox patients with ordinary STDs without thoroughly considering the range of other possible differential diagnoses. Some of the outbreak’s less frequently reported cases may also be due to the less severe clinical symptoms associated with clade 2 MPXV. Patients who feel good might also not seek medical attention (Patrono et al., 2020; Jonge et al., 2022; Nörz et al., 2022; Peiró-Mestres et al., 2022; Pfeiffer et al., 2022; Sharkey et al., 2022; Atkinson et al., 2022a,b; CDC, 2023d).

Semen samples from asymptomatic people in this outbreak contained MPXV DNA, suggesting hat asymptomatic transmission may have contributed to the virus’s spread. In a recent investigation, rectal swabs from 200 asymptomatic MSM cases revealed positive Mpox qPCR results. To establish the presence of a contagious virus, isolating it from tested samples is necessary because qPCR results cannot precisely predict the chance of infection. The existence of asymptomatic Mpox cases raises concerns that the true number of patients may be underestimated and that the virus may be spread to close contacts even when there are no symptoms. Collecting more accurate epidemiologic data on the prevalence, clinical signs, transmission mechanism, and asymptomatic cases in the general population and individuals with other STDs is necessary (Patrono et al., 2020; Jonge et al., 2022; Peiró-Mestres et al., 2022; Sharkey et al., 2022; Wurtzer et al., 2022; Gazecka et al., 2023; Tiwari et al., 2023; Wannigama et al., 2023; CDC, 2023d).

## 3 Environmental contamination of MPXV

### 3.1 MPXV persistence on surfaces in healthcare settings

The contamination of healthcare settings by MPXV and its ability to cause infection is also vital when dealing with the recent outbreak, significantly helping protect healthcare providers from getting infected (Nakoune et al., 2017). Healthcare professionals are a high-risk group for getting infected and infecting others as they are exposed to many patients daily.

Few cases of healthcare-associated human Mpox have been recorded in endemic African nations throughout history. These cases were documented in the Democratic Republic of the Congo in 1983 (Ježek et al., 1986), the Republic of the Congo in 2003 (Learned et al., 2005), the Central African Republic in 2015 and 2016 (Nakoune et al., 2017), and Nigeria in 2017 and 2018 (Ogoina et al., 2019). Although all these exposures took place in hospital Settings, the precise routes of spread could not be determined. Between 2003 and 2021, at least 250 healthcare professionals had varying levels of unprotected contact with MPXV while working in a hospital environment. Yet, only one incidence of nosocomial transmission is documented in the literature (Vaughan et al., 2020; Zachary and Shenoy, 2022). That case report represented a healthcare worker identified as having Mpox in the UK in 2018 and whose only known exposure risk was replacing a patient’s possibly contaminated bedding (Vaughan et al., 2020).

Further, fomite transmission has been reported as a single route of transmission of Mpox in two cases out of 3,924 cases (Salvato et al., 2022). In this study, two cases of fomite transmission involved healthcare workers who spent an hour at the patient’s home, wore PPE (N95 masks, eye protection, gowns), only used gloves when taking clinical samples from patients, and avoided direct physical contact with the patient. Authors reported that Mpox could have been transmitted exclusively through fomites in those two cases in contrast to 2,420 out of 3,924 cases with direct physical or sexual contact as the transmission mode (Salvato et al., 2022). Although indirect transmission through contaminated objects or surfaces has been reported, the evidence of fomite transmission for Mpox is currently limited (Vaughan et al., 2020; Kozlov, 2022). However, it is still a rare method of transmission. Other transmission methods are already considered, and variable preventive measures are applied for their prevention.

After demonstrating the evidence of the possibility of nosocomial transmission and evidence of surface contamination at the homes of the infected persons, the question of whether surface contamination in hospital settings and whether contaminated objects are a cause of Mpox dissemination is one the scientific community is preoccupied with (Kozlov, 2022; Ogoina and Ogunsola, 2022).

A study conducted in Germany evaluated the contamination of surfaces in hospital rooms and found that the most significant virus loads were found in toilets. However, contamination was present on all surfaces contacted directly by the patients. It was also detected on the patients’ mobile phones, toilet seats, and chair seat surfaces (Nörz et al., 2022). Fabrics used frequently by the patients were also contaminated with viruses. After touching these fabrics by investigators, the investigator’s gloves were swabbed on the palmar side, and the contamination on that side was determined. Interestingly, these samples showed positive culture, which supports the evidence of infection from touching and objects used or touched by any infected person. In addition, all hand-contact sites, such as the door handle, in the anteroom that were investigated showed positive qPCR findings (CDC, 2003b; Ogoina et al., 2019; Vaughan et al., 2020; Gould et al., 2022; Morgan et al., 2022; Nörz et al., 2022; Ogoina and Ogunsola, 2022; Pfeiffer et al., 2022; Zachary and Shenoy, 2022).

A more extensive study in a UK hospital (Gould et al., 2022) found MPXV DNA in 56 (93%) of 60 surface swab samples taken from patient bedrooms and toilets. Some of the samples that tested positive were from places patients were not likely to have touched directly, such as the air vent over the bathroom door and the air vent above the bedroom door, indicating non-contact contamination, presumably through respiratory droplets or particles suspended in the air after changing bedding.

The DNA of the virus was also detected from two samples swapped from the gloves. The discovery of the contagious MPXV in air samples taken during a bedding change emphasizes the necessity of respiratory protection for healthcare professionals while conducting activities that could contain infectious material in contaminated settings (Ježek et al., 1986; CDC, 2003b; Nakoune et al., 2017; Ogoina et al., 2019; Vaughan et al., 2020; Ferré et al., 2022; Gould et al., 2022; Morgan et al., 2022; Nörz et al., 2022; Ogoina and Ogunsola, 2022; Ogoina and Yinka-Ogunleye, 2022; Pfeiffer et al., 2022; Zachary and Shenoy, 2022; Atkinson et al., 2022b).

There was variability in the frequency of virus detection from one patient’s room to another. That can be attributed to disease severity as the extent of contamination varies depending on viral load. Patients suffering from severe illness are more likely to have a higher viral load, leading to increased shedding and potentially higher environmental contamination. Also, the period during the patient’s illness when environmental sampling was done, staff or patient behavior, and Infection control measures. However, no variation in cleaning procedures might account for the variations in environmental sample data, and none of the patients’ clinical features can account for the variations in the findings of the air sampling for the various isolation rooms examined.

Other factors might have accounted for this variability, necessitating further investigation (Ježek et al., 1986; CDC, 2003b; Nakoune et al., 2017; Ogoina et al., 2019; Vaughan et al., 2020; Ferré et al., 2022; Gould et al., 2022; Morgan et al., 2022; Nörz et al., 2022; Ogoina and Ogunsola, 2022; Pfeiffer et al., 2022; Zachary and Shenoy, 2022; Atkinson et al., 2022b).

In a cross-sectional study, Hernaez et al. (2023) assessed the presence of MPXV DNA in saliva, exhaled droplets from a mask, and aerosols from patients with qPCR-confirmed Mpox infection attending two healthcare centers in Spain. They reported high viral load detected by qPCR in saliva samples and viable virus in 66% of qPCR-positive saliva samples. These findings signify that saliva might assist in contaminating surfaces with the infectious virus, respiratory droplets, aerosols. According to studies above (Gould et al., 2022; Nörz et al., 2022), MPXV DNA has been found by qPCR on various surfaces in hospital rooms occupied by Mpox patients. Some contained the contagious virus, which the previous findings could explain. The exact persistence of MPXV in healthcare settings and its availability to cause infection is not identified definitively. However, the evidence of its persistence for 15 days in a home setting is alarming and should increase our attention (Morgan et al., 2022). Thus, measuring its infectivity power and its existence raises a major concern. Numerous variables might affect a human being successfully being infected with a virus; therefore, just because a virus is detected in environmental samples does not always indicate that transmission resulting in infection would occur if that virus were exposed to a person (Gould et al., 2022). These variables include modes of transmission, host susceptibility, environmental elements that may impair the virus capacity to replicate and infect cells, and the quantity of virus to which a person is exposed (Gould et al., 2022). The infectious dosage ofMPXV in people is unknown and may vary depending on the body part exposed to the virus.

Various lessons should be considered during this outbreak to protect healthcare providers and prevent the spread of the virus in hospital settings. Firstly, the pervasive surface contamination of the patient care environment necessitates a methodical, standard approach to surface disinfection of hospital settings and homes of patients with Mpox. Secondly, the discovery of MPXV DNA on personal protective equipment (PPE), in doffing areas, and air samples were taken at different distances from the patient bed and during bed sheet changes emphasizes the significance of using and removing PPE properly by healthcare professionals to prevent exposure to Mpox while providing patient care (Gould et al., 2022).

Third, since changing bedding may spread MPXV particles, staff members should wear surgical masks (with or without a face shield) at the very least to protect their mucous membranes (CDC, 2003b; Gould et al., 2022; Ogoina and Ogunsola, 2022). Therefore, disinfectants must be used for sites of isolation of Mpox patients.

### 3.2 MPXV persistence on surfaces in households

Surface contamination may contribute to the spread of infection and is therefore essential to study, especially in the presence of asymptomatic cases that may contribute to virus transmission. Several studies have been conducted to measure MPXV contamination on different surfaces (Nörz et al., 2022; Pfeiffer et al., 2022; Atkinson et al., 2022b). Despite this, little is known about the extent of surface contamination by MPXV. There are several gaps in knowledge, such as the duration of contamination of different surfaces, and further studies are necessary to provide more detailed and accurate information.

CDC and the Utah Department of Health and Human Services (UDHHS) investigated MPXV surface contamination on frequently used household surfaces in May 2022 (Pfeiffer et al., 2022). Patients were still symptomatic at the time of sample collection, obtained from 9 different locations throughout the house and examined using qPCR. Of them, 21 of the 30 samples from porous objects tested positive for Mpox, and 17 of 25 non-porous items developed positive qPCR results. Fabric surfaces such as blankets and chaise lounge tested, and hard surfaces such as light handles and a keyboard tested positive. However, none of the surfaces that tested positive by qPCR yielded a positive viral culture test. It is suggested that cleaning or passing of time may have affected the viral load and inactivated the virus (Ježek et al., 1986; CDC, 2003b; Ogoina et al., 2019; Vaughan et al., 2020; Gould et al., 2022; Morgan et al., 2022; Nörz et al., 2022; Ogoina and Ogunsola, 2022; Zachary and Shenoy, 2022).

Additionally, environmental sampling was carried out in the house of a resident in the USA who had confirmed infection with the west African clade after travelling to Nigeria 15 days after the patient left the residence. Results suggested substantial MPXV DNA contamination as seven samples yielded viable viruses isolated in cell culture. That study demonstrated no difference in qPCR positivity between porous and non-porous surfaces but noted a significant difference in the virus detected in cultures. That suggests that porous surfaces may pose a higher risk of Mpox infection (Morgan et al., 2022).

In June 2022, another study investigated the presence and extent of surface contamination in the hospital rooms of two patients infected with MPXV on the fourth hospitalization day. All surfaces touched by both patients showed viral contamination, with the highest detected on bathroom surfaces. According to the number of viral copies per cm^2^ in the first patient, the highest viral load was found on the tap control lever, the seating surface toilet seat, and the mattress cover. In the second, the highest viral load was found on the towel in bed, soap dispenser lever, and pillowcase used to cover cooling packs, followed by the glove used by the examiner. After handling fabrics, the examiners’ gloves were immediately swabbed from the palmer side and found to have contamination levels in both patients (Nörz et al., 2022). The investigators had successfully isolated MPXV using three different samples, each containing a minimum of 10^6^ virus copies. As a result, contaminated surfaces that carry such viral loads or higher may be contagious (Pfeiffer et al., 2022; Atkinson et al., 2022a; CDC, 2023d).

The CDC in the United States of America has advised that close contacts of Mpox patients should watch for any MPXV symptoms for 3 weeks following the last exposure. In addition, the CDC recommended getting vaccinated and seeking medical attention if an unexplained rash emerged after touching an infected person. Sharing dishes, towels, bed sheets, clothes, drinking glasses, or other private belongings is also not advised (CDC, 2003b; Brown and Leggat, 2016; Patrono et al., 2020; Dye and Kraemer, 2022; Gould et al., 2022; Jonge et al., 2022; Morgan et al., 2022; Nörz et al., 2022; Ogoina and Ogunsola, 2022; Peiró-Mestres et al., 2022; Pfeiffer et al., 2022; Sharkey et al., 2022; Wurtzer et al., 2022; Atkinson et al., 2022a,b; Gazecka et al., 2023; Tiwari et al., 2023; Wannigama et al., 2023; CDC, 2023d). It is strongly recommended to disinfect surfaces, and this should be done with cleaning supplies from the CDC and Environmental Protection Agency lists to prevent cross-contamination. Disposable gloves, a face mask, and clothing covering their arms and legs are all recommended when disinfecting. These suggestions are examples of ways to stop the transmission of Mpox; however, other nations may have different laws; therefore, they may not apply there (Klein, 1963; Kampf et al., 2002; Wutzler and Sauerbrei, 2004; Kampf et al., 2007; Rabenau et al., 2010; Steinmann et al., 2012; Brown and Leggat, 2016; Petersen et al., 2016; Becker et al., 2017; Siddharta et al., 2017; Dye and Kraemer, 2022; Ogoina and Ogunsola, 2022; Upadhayay et al., 2022).

### 3.3 MPXV contaminating wastewater

Another concern was the contamination of wastewater. It is not fully known how MPXV ends up in sewage. Excretion and secretion of MPXV through faeces were observed in infected animals and humans (Patrono et al., 2020; Peiró-Mestres et al., 2022). Another plausible transmission mode is by skin flakes from affected body areas that wash into wastewater; this is supported by the high viral concentration found in blisters and scabs (Jonge et al., 2022).

According to data from various body fluids, viral shedding can occur from several body sites, including saliva, semen, urine, and faeces. That suggests that bodily fluids may play a part in the spread of disease (Peiró-Mestres et al., 2022).

To check for the existence of MPXV nucleic acids, a team of researchers examined the presence of DNA and RNA extracts from wastewater. Viral particles were investigated by examining the presence of packaged DNA in the viral capsid. Wastewater included human beta-2-microglobulin (B2M) RNA, indicating that excretion and washing can cause the nucleic acids found in human cells to be discovered in sewage (Jonge et al., 2022; Ogoina and Ogunsola, 2022; Peiró-Mestres et al., 2022; Sharkey et al., 2022; Wurtzer et al., 2022; Gazecka et al., 2023; Tiwari et al., 2023). Midway through July, MPXV was found in the wastewater treatment facility for the first time. Over time, higher quantities were observed. Data also revealed a correlation between wastewater levels and the amount of reported clinical cases (Brown and Leggat, 2016; Petersen et al., 2016; Dye and Kraemer, 2022; Ogoina and Ogunsola, 2022; Tiwari et al., 2023).

In addition, a recent study in Amsterdam detected MPXV DNA in wastewater samples from two wastewater treatment facilities, Schiphol Airport, and five different city districts. The sample was deemed positive if both the generic and west-African qPCR were present. Out of 108 wastewater samples, MPXV DNA was discovered in 45 of them. Because infected people may have passed through, samples from the airport occasionally tested positive for MPXV DNA. A sporadic rise in the number of positive samples taken in Schiphol was associated with an increase in confirmed cases. That is consistent with the study’s findings described earlier. To verify the DNA specificity, qPCR and an extra traditional qPCR were performed on a predetermined subset of the obtained samples (Klein, 1963; Kampf et al., 2002; Wutzler and Sauerbrei, 2004; Kampf et al., 2007; McDevitt et al., 2007; CDC, 2008; McDevitt et al., 2008; Russell, 2008; Eterpi et al., 2009; Rabenau et al., 2010; de Oliveira et al., 2011; Steinmann et al., 2012; Bleichert et al., 2014; Eggers et al., 2015; Brown and Leggat, 2016; Campagna et al., 2016; Petersen et al., 2016; Becker et al., 2017; Siddharta et al., 2017; Dye and Kraemer, 2022; Ogoina and Ogunsola, 2022; Upadhayay et al., 2022; Meister et al., 2023).

Researchers collected wastewater samples between May and August 2022 from 63 sewered and non-sewered places in Bangkok’s city centre. qPCR was used to measure the MPXV DNA copy counts, and Sanger sequencing was used to validate the results as positive. Beginning in the second week of June 2022, wastewater samples containing MPXV DNA had a mean copy number of 16.4 copies/mL. Sanger sequencing of positive samples confirmed the MPXV’s existence. According to preliminary analyses, the MPXV DNA from wastewater samples also belonged to the West African lineage. All places where MPXV DNA was detected had closed, un-severed sewage systems and were either public or commercial (Wannigama et al., 2023).

Investigators in Spain found MPXV DNA in 56 of 312 wastewater samples they took from various country locations. Their CT scores varied from 39.98 to 34.5. Interestingly, samples taken from Madrid WWTPs during week 23 of 2022, when just 275 cumulative cases had been reported across the entire region, consistently contained MPXV DNA. Additionally, some WWTPs with limited confirmed clinical patients reported intermittent detection (Girón-Guzmán et al., 2023). That could mean that silent infections are occurring more frequently than anticipated and that the reported clinical cases appear to be underestimated. To estimate the actual number of Mpox cases, wastewater monitoring is crucial during an outbreak. Another Polish study that revealed no relationship between the quantity of hospitalized patients in a given area and MPXV detection in WWTPs supports this result (Gazecka et al., 2023). That is in contrast to a study that found that the discovery and quantification of the MPXV genome in sewersheds in Paris coincided temporally with the discovery of the first case of infection and the spread of the disease throughout the population connected to the sewage system (Wurtzer et al., 2022).

Wastewater surveillance is a well-established supplemental epidemiologic tool that has been effectively employed for viral infectious illnesses, including SARS-CoV-2 and polio; therefore, investigating potential strategies for monitoring MPXV through these systems is crucial. Three distinct PCR tests that were previously developed for clinical samples were put to the test by a research team. To lessen the impact of nucleotide mismatches, they altered the tests by making alterations to the primer and probe sequences. Using real-time or nested PCR and sequencing, three samples out of 20 tested positive for MPXV, demonstrating that these techniques can be used for wastewater-based epidemiology for Mpox outbreaks and offering fundamental resources, such as analytical techniques (La Rosa et al., 2023; Tiwari et al., 2023).

Untreated wastewater can track the movement and dispersal of many diseases. As it contains a variety of biological materials, such as skin, vesicular fluid, saliva, semen, faeces, and respiratory and nasal secretions, it is perfect for monitoring. A customizable platform called wastewater-based surveillance (WBS) can show in real-time when infectious pathogens are being shed; genetic material may be discovered days before symptoms or a healthcare facility’s confirmation of infection. WBS has advantages and disadvantages; benefits include cost-effectiveness, independence from testing capacity, patient permission to test, and data utilization by third parties. Disadvantages include the absence of standardization and interference from different substances; since WBE is not always sensitive enough to pick up minute amounts of pathogens, it might be challenging to obtain reliable results from wastewater samples (Gerba et al., 1980; Farahbakhsh and Smith, 2004; John and Rose, 2005; McDevitt et al., 2007; CDC, 2008; McDevitt et al., 2008; Russell, 2008; Gundy et al., 2009; de Oliveira et al., 2011; Bleichert et al., 2014; Campagna et al., 2016; Sassi et al., 2018; Meister et al., 2023).

Detection of MPXVs in wastewater depends on many variables, including infection rate, water flow, viral shedding, and the process used for detection and analysis. Cross-reaction between the assay and non-targeted pathogens is a challenging possibility as wastewater contains many different microbes from multiple sources. The most concerning challenge with WBS is the lack of standard procedures and methodology used to collect samples, measure viral concentrations, extract DNA, and interpret data. Due to the scarcity of essential data on Mpox infection, it’s challenging to create a prediction model or to establish a correlation between reported clinical cases and available WBS data. Further research is necessary to fill in critical knowledge gaps, such as the persistence of MPXV in different environments, MPXV DNA shedding in other body fluids, and the extent of MPXV infectivity in wastewater. Filling in these gaps is necessary to quickly develop a robust global WBS network (Klein, 1963; Wutzler and Sauerbrei, 2004; McDevitt et al., 2007, 2008; Eterpi et al., 2009; Rabenau et al., 2010; de Oliveira et al., 2011; Bleichert et al., 2014; Eggers et al., 2015; Campagna et al., 2016; Meister et al., 2023).

## 4 Controlling Mpox transmission

### 4.1 Disinfection of surfaces

The use of disinfectants to eliminate MPXV from the surfaces and environment is now considered a vital issue after proving its presence on the surfaces and in the environment and its possibility to cause infection (Gould et al., 2022; Nörz et al., 2022; Ogoina and Ogunsola, 2022; Atkinson et al., 2022a). Therefore, disinfection around the confirmed cases may be necessary to lessen the risk of viral transmission via contaminated surfaces. Consequently, it is crucial to understand which disinfectants and biocidal chemicals are efficient against MPXV and other orthopoxviruses, even though it is doubtful that mpox would cause a worldwide health disaster (Dye and Kraemer, 2022).

Vaccinia virus has been extensively studied and used as a surrogate for other orthopoxviruses, including MPXV, due to its availability, ease of handling, and well-established protocols for inactivation an disinfection (Brown and Leggat, 2016). Many disinfection protocols and guidelines for orthopoxviruses, including those recommended by the World Health Organization, are based on studies using vaccinia virus as a surrogate (Petersen et al., 2016). While there may be some differences between the vaccinia virus and MPXV in their behavior in certain disinfection conditions (Upadhayay et al., 2022), vaccinia virus as a surrogate is generally considered appropriate, especially when primary data for MPXV is unavailable.

Ethanol (ranging 50%–90%) proved effective in suspension experiments against the vaccinia virus strain Elstree and the modified vaccinia virus Ankara (MVA) at concentrations ranging from 50% to 95% within 1 min, even with varied organic loads (Kampf et al., 2002, 2007; Steinmann et al., 2012; Siddharta et al., 2017). Phosphoric acid added to ethanol at 45% also worked for 30 s. However, ethanol only showed marginal effectiveness in doses of 40% or less (Steinmann et al., 2012). Formulas containing two kinds of alcohol and a combined alcohol content between 75% and 77.8% were likewise quite effective in 15 s (Kampf et al., 2002, 2007).

Peroxides also showed good effectiveness against the vaccinia virus (Kampf et al., 2002). In suspension experiments, hydrogen peroxide proved efficient against the vaccinia virus at 14.4% concentration in 30 s (Wutzler and Sauerbrei, 2004; Rabenau et al., 2010; Becker et al., 2017). Additionally, it was shown that peracetic acid rapidly rendered vaccinia viruses inactive at concentrations between 0.005 and 0.2% within 1 min and with 10% fetal calf serum (FCS) as an organic load (Rabenau et al., 2010; Becker et al., 2017). Glutaraldehyde was successful in suspension tests against the vaccinia virus strains Elstree and MVA at concentrations between 0.05 and 0.5% within 5 min, often in the presence of 10% FCS (Klein, 1963; Rabenau et al., 2010). However, significant activity against vaccinia viruses was not documented at shorter contact periods of 30 s or 2 min (Klein, 1963; Rabenau et al., 2010).

With a low organic load, chlorine proved effective against vaccinia viruses in suspension testing at 0.64% in 1 min and 0.525% in 3 min (Eterpi et al., 2009; Eggers et al., 2015). Lower concentrations either needed longer exposure durations or were not powerful enough. However, increased albumin content as an organic load decreased the virucidal effectiveness (Eterpi et al., 2009). Under clean and unclean test circumstances, iodine was also potent against vaccinia viruses at concentrations between 0.045 and 1% within 1 min (de Oliveira et al., 2011; Eggers et al., 2015). However, under dirty test circumstances on artificially contaminated stainless-steel carriers, sodium hypochlorite (0.25% and 2.5%) proved also efficient against the vaccinia virus in one minute (Eterpi et al., 2009; de Oliveira et al., 2011; Eggers et al., 2015).

An alkaline cleanser at 0.9% was shown to inactivate the vaccinia virus in 10 min under unclean conditions on artificially polluted stainless-steel carriers (Eterpi et al., 2009). Ultraviolet light (254 nm) has been shown to inactivate aerosolized vaccinia virus strain WR in a tabletop one-pass aerosol chamber in 7.6 s (McDevitt et al., 2007, 2008).

The vaccinia virus was more vulnerable to UVC with decreased relative air humidity (McDevitt et al., 2007). Similar outcomes were seen when the Western Reserve strain of the vaccinia virus was exposed to UVC light for 10 min through aerosol (254 nm) (McDevitt et al., 2007, 2008).

In summary, vaccinia viruses could be rendered inactive by a 1 min application of 70% ethanol, 0.25% and 2.5% sodium hypochlorite, 14.4% hydrogen peroxide, 0.64% chlorine, and 0.045 and 1% iodine. It also can be killed by a 3 min application of 99.9 copper or 0.525% chlorine, 5 min of 0.55% ortho-phthalaldehyde, 10 min of 2% glutaraldehyde, 0.2% peracetic acid, 0.9% alkaline cleanser, or 254 nm ultraviolet light. Few studies investigated the efficacy of some disinfectants on Mpox. For example, one study found that in 3 min, copper with a purity of 99.9% was as effective against the vaccinia virus as it was against MPXV (Bleichert et al., 2014). Another study on the hand rub formulations and alcohols recommended by the WHO found that, despite these disinfectants being effective, MPXV showed the greatest stability compared with other enveloped viruses (Meister et al., 2023). It is crucial to note that the efficacy of a disinfectant against MPXV can be influenced by parameters such as the kind and amount of organic material present, disinfectant concentration and contact time, and environmental temperature and humidity (CDC, 2008; Russell, 2008; Campagna et al., 2016). However, we recommend validating disinfection protocols using MPXV to ensure their effectiveness in real-world settings.

### 4.2 Treatment of wastewater

Treatment of wastewater is a crucial step to avoid the further spread of diseases. Conventional treatment plants consist of a primary sedimentation step, secondary aerobic treatment and chlorination before reuse and discharge. The survival of viruses is mainly dependent on temperature; high temperatures denature proteins and increase enzymatic activity, decreasing viral survival rates (Gerba et al., 1980; John and Rose, 2005). Solvents and detergents also play a role as they remove viral envelopes. Anaerobic treatment denatures proteins and nucleic acids (Gundy et al., 2009; Sassi et al., 2018).

Removal of viruses is usually greater in the secondary treatment plant; this is attributed to sludge and suspended solids in the secondary effluent to which viruses attach. Viruses are mainly adsorbed in the second treatment (John and Rose, 2005). Viral inactivation especially takes place in tertiary treatment, which is the most crucial step to stop the transmission of infection. Advanced treatment is membrane separation, used in water reuse. It also plays a role in removing viruses from wastewater. A study revealed that using foul membranes for microfiltration removed more viruses than clean membranes (Farahbakhsh and Smith, 2004).

The currently implemented wastewater treatment systems are adequate at removing MPXV from wastewater and helping prevent the spread of infection. However, further studies may be needed to provide more information regarding the optimal temperature and best-suited disinfectant to rid wastewater from MPXV completely.

## 5 Conclusion

Current evidence demonstrates the contamination of surfaces by infected persons with mpox that has been proven to be stable and viable in the environment, surviving for a varying time depending on factors that impact virus survival, such as the contaminated surface, humidity, and temperature. This is to be emphasized that positive qPCR results do not necessarily means viral infectivity. It is essential to keep in mind that indirect transmission of mpox through contaminated surfaces may present a significant issue for international health organizations by highlighting the risks of long-distance transmission, bringing back the virus into an area that has attained regional elimination with extended outbreak duration. Due to asymptomatic cases, it is currently more challenging to identify the infection’s origin and spread. Thus, personal protective measures, such as hand washing and regular disinfection, should reduce environmental contamination and the possibility of virus transmission.

Wastewater surveillance is imperative in developing systems for efficient, timely control of diseases. However, poor urban planning structures, growing populations, and transportation challenges worsen the wastewater treatment problem in these countries. As a result, appropriate wastewater treatment services must be established.

In summary, focusing on interventions related to the highest-risk-associated settings for mpox infection spread and transmission is an effective strategy for preventing and reducing the risk of mpox spread. Furthermore, our findings could form the base for future research into mpox transmission and persistence in the environment. It assists in preventing and reducing the negative consequences of this disease. Further research is needed to fill in knowledge gaps, to help understand the persistence of the virus on surfaces in healthcare settings and the environment, viral shedding and DNA levels in different body fluids and skin lesions, and the persistence of MPXV DNA and its infectivity in wastewater. Improvements in the analytical methods used to detect MPXV DNA in sewage are also needed.

## Author contributions

AT: Conceptualization, Writing – original draft, Writing – review & editing. BK: Conceptualization, Writing – original draft, Writing – review & editing. AH: Conceptualization, Writing – original draft, Writing – review & editing. OA: Conceptualization, Writing – original draft, Writing – review & editing. SR: Conceptualization, Writing – original draft, Writing – review & editing. DN: Conceptualization, Writing – original draft, Writing – review & editing. KS-C: Writing – review & editing. AR-M: Writing – review & editing. AM: Writing – review & editing. DB-A: Writing – review & editing. RS: Writing – review & editing.

## Figures and Tables

**Table 1:** 

qPCR	Quantitative polymerase chain reaction
WBS	Wastewater-based surveillance
DNA	Double-stranded nucleic acid
RNA	Ribonucleic acid
MPXV	Monkeypox virus
RT-PCR	Reverse transcription polymerase chain reaction
UV	Ultraviolet
CDC	Centers for Disease Control and Prevention
UK	United Kingdom
STD	Sexually transmitted diseases
MVA	Modified vaccinia virus Ankara
FCS	Fetal calf serum
B2M	Beta-2-microglobulin
V2G	Volcano 2nd generation
PPE	Personal protective equipment
MSM	Men who have sex with men
SARS-CoV-2	Severe acute respiratory syndrome coronavirus 2
